# Supplementary figures and images for: Collagen Family and Other Matrix Remodeling Proteins Identified by Bioinformatics Analysis as Hub Genes Involved in Gastric Cancer Progression and Prognosis
Source: Int J Mol Sci. 2022 Mar 16;23(6):3214. doi: 10.3390/ijms23063214 (PMC8950589; doi:10.3390/ijms23063214)

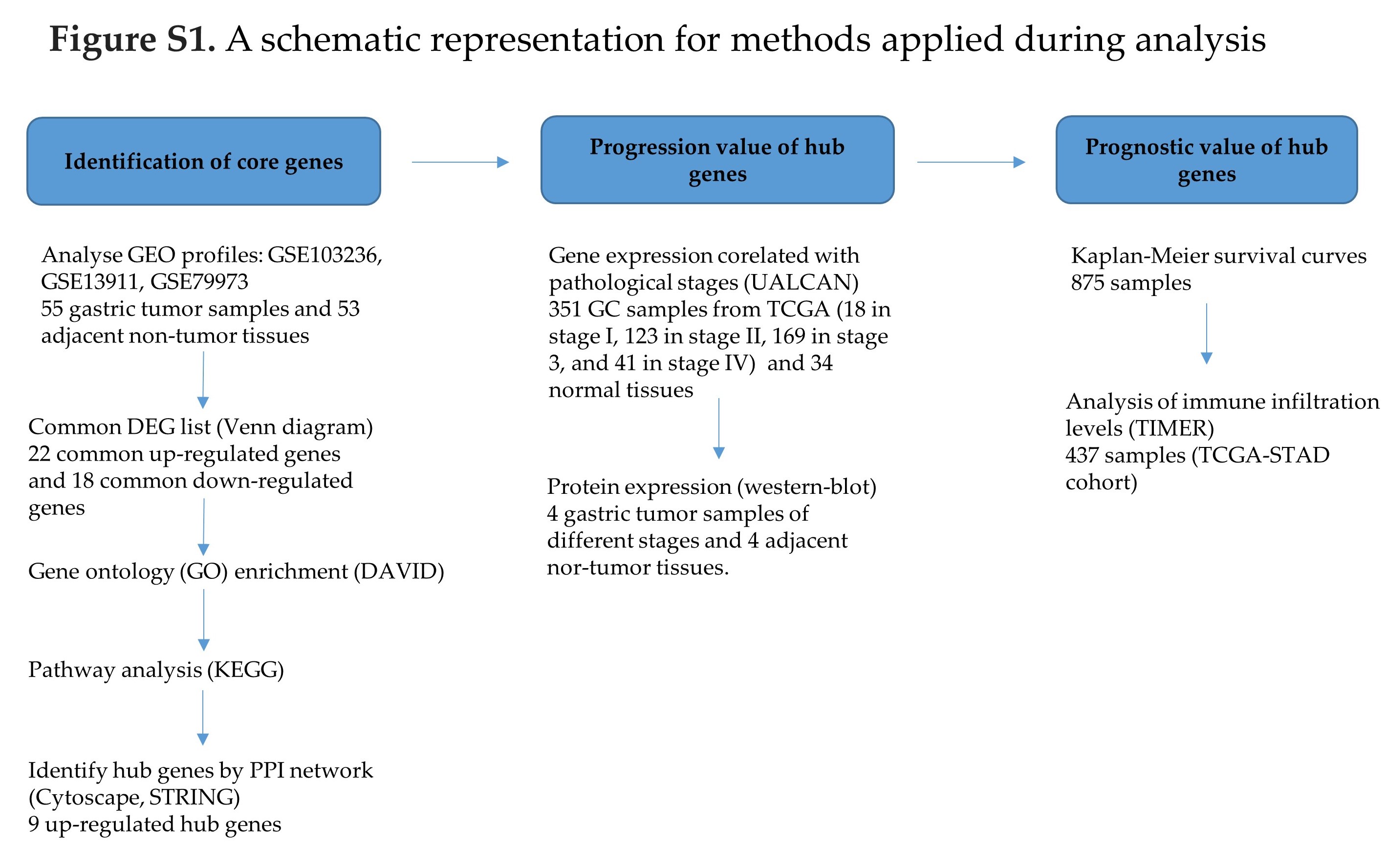

Supplement: Supplementary file 1 [file ijms-23-03214-s001.zip › ijms-1629527-supplementary/Figure S1.jpg]
